# Microplastic in Dredged Sediments: From Databases to Strategic Responses

**DOI:** 10.1007/s00128-024-03878-x

**Published:** 2024-04-30

**Authors:** J. L. Wilkens, A. J. Calomeni-Eck, J. Boyda, A. Kennedy, A. D. McQueen

**Affiliations:** Engineer Research and Development Center, US Army Corps of Engineers, 3909 Halls Ferry Rd, Vicksburg, MS 39180 USA

**Keywords:** Plastic particles, Contamination, Marine, Freshwater, Sediment, Dredging

## Abstract

Microplastics (MPs) accumulate in sediments, yet guidelines for evaluating MP risks in dredged sediments are lacking. The objective of this study was to review existing literature on MPs in sediments to improve fundamental knowledge of MP exposures and develop a publicly available database of MPs in sediments. Twelve percent of the reviewed papers (nine studies) included sediment core samples with MP concentrations generally decreasing with depth, peaking in the top 15 cm. The remaining papers evaluated surficial grab samples (0 to 15 cm depth) from various water bodies with MPs detected in almost every sample. Median MP concentrations (items/kg dry sediment) increased in this order: lakes and reservoirs (184), estuarine (263), Great Lakes nearshore areas and tributaries (290), riverine (410), nearshore marine areas (487), dredge activities (817), and harbors (948). Dredging of recurrent shoaling sediments could be expected to contain MPs at various depths with concentrations influenced by the time elapsed since the last dredging event. These results offer key insights into the presence and variability of MPs in dredged sediments, informing environmental monitoring and risk assessment strategies.

Microplastics (MPs), ranging from 0.01 to 5 mm (Frias and Nash 2019), are ubiquitous in sediments, serving as environmental sinks with potential for bioaccumulation in ecosystems and possibly in humans (Darabi et al. 2021). Their small size and ability to degrade into nanoplastics is cause for concern about ingestion by wildlife and the resultant ecological risks, which has gained international (UN 2022) and national attention (USEPA 2016). The US Army Corps of Engineers (USACE) confronts this directly, annually dredging several hundred million cubic yards of sediment each year, often without data on MPs abundance in shoaled channel beds requiring dredging. The International Maritime Organization, a United Nations (UN) specialized agency, and the Group of Experts on the Scientific Aspects of Marine Environmental Protection, a UN advisory body, both recognize that plastics are in shoaled sediments destined for dredging and the challenges associated with separating plastic from dredged sediment (IMO 2016; GESAMP 2021). Recognizing the need for informed decision-making in the management of dredged sediments, the primary objective of this study is to synthesize existing peer-reviewed research to provide a comprehensive understanding of the presence, concentration, and characteristics of MPs in sediments, especially those that may be relevant to national and international navigation channels requiring periodic dredging. By presenting MP concentrations, morphologies, size ranges, colors, and polymer types, we aim to provide project managers a foundational reference that serves as a starting point for a risk-informed response strategy for dredged sediments that balances environmental risks and operational demands.

## Methods and Materials

In March 2023, we systematically searched Web of Science and Google Scholar for studies on MPs in dredged sediments, focusing on sediment collected during dredge sediment evaluations or sediment collected from disposal areas (search “microplastics dredging dredge”). The scarcity of research specifically on dredged sediments broadened our scope to similar environments, such as harbors, nearshore marine and estuarine areas (subtidal), as well as riverine and lacustrine systems including the Great Lakes (search “microplastics sediments”). These areas were likely to share similar plastic inputs and physical processes as would take place for shoaled sediment destined for dredging. We refined the collected literature against minimum data quality objectives, prioritizing studies that detailed their MP detection methods, utilized visual and/or spectrometric identification, and reported MP concentration per kilogram of dry sediment. When necessary (n = 4 studies), MP quantities reported as MP per liter were standardized to MP per kilogram dry sediment following the methodology of Claessens et al. (2011), to allow for more direct comparison across studies. In this paper, MP concentrations are reported as “items/kg” used to encompasses a variety of MP morphologies (fibers, films, foams, fragments, spheres or beads) and polymer types.

For each study, data was disaggregated by specific test areas within larger water bodies when such information was available. Our exploratory analysis focused on presenting median MP concentrations, a metric robust against skewed distributions, along with the full range of MP concentrations (minimum to maximum) to encapsulate the heterogeneity in environmental conditions as well as sampling and laboratory methodologies. To offer a more granular perspective, we categorized sample locations into systems including lakes, rivers, the Great Lakes, estuarine and marine environments, harbors and ports, and studies specifically addressing dredging. For instances of notably high or low MP abundances, additional contextual information is provided to elucidate possible environmental or methodological factors contributing to these extremes.

## Results and Discussion

The review identified 122 papers, with 73 meeting our priorities based on their relevance and methodological clarity. Data from this review was collated in an online microplastic database for sediments (MP-SED 2023) which contains a searchable database of MP concentrations, sizes, shapes, and polymer data for sediments across diverse geographic locations. These reviewed papers, spanning 2004 to 2023 and retrieved from 31 journals, predominantly featured MP concentrations in sediment cores and in the top 0–15 cm of sediments collected in North America (30%), Europe (31%), and Asia (32%), with minor contributions from Africa and Oceania. In our review, data extraction from the papers directly yielded MP data from tables in 63% of the of the papers reviewed. For the remainder of the papers reviewed, MP data required interpolation from figures using image analysis software (ImageJ, version 1.54c, NIH, Bethesda, MD). Overall, MPs less than 1–2 mm in size were the most abundant. Fibers and fragments were the dominant morphologies, and occasionally spheres and foams, and to a much lesser extent, films. Commonly observed colors included black, blue, brown, white, gray, red, yellow, green, pink, purple, orange, and transparent. Plastics consisting of polypropylene, polyethylene, polyethylene terephthalate, Rayon, Nylon, polystyrene, and tire wear particles were most identified. The focus on MPs themselves often leads researchers to overlook sediment characteristics, such as sand, silt, and clay, which are not reported in many papers cited here. Although some research has explored the correlation between sediment types and MP concentrations, the results have varied (Alomar et al. 2016; Corcoran et al. 2020). Data from sediment cores are presented below to understand the potential presence and concentration of MPs at various depths. For grab samples, MPs in the top 0–15 cm of sediments are presented for each previously listed system (e.g., Great Lakes, riverine, harbors and ports). Further, contextual information is provided to understand potential MP contamination during capital dredging which entails deeper excavation (i.e., several meters) of bottom sediments and during maintenance dredging (~ top meter of sediment), the most common form of dredging.

**Fig. 1:**
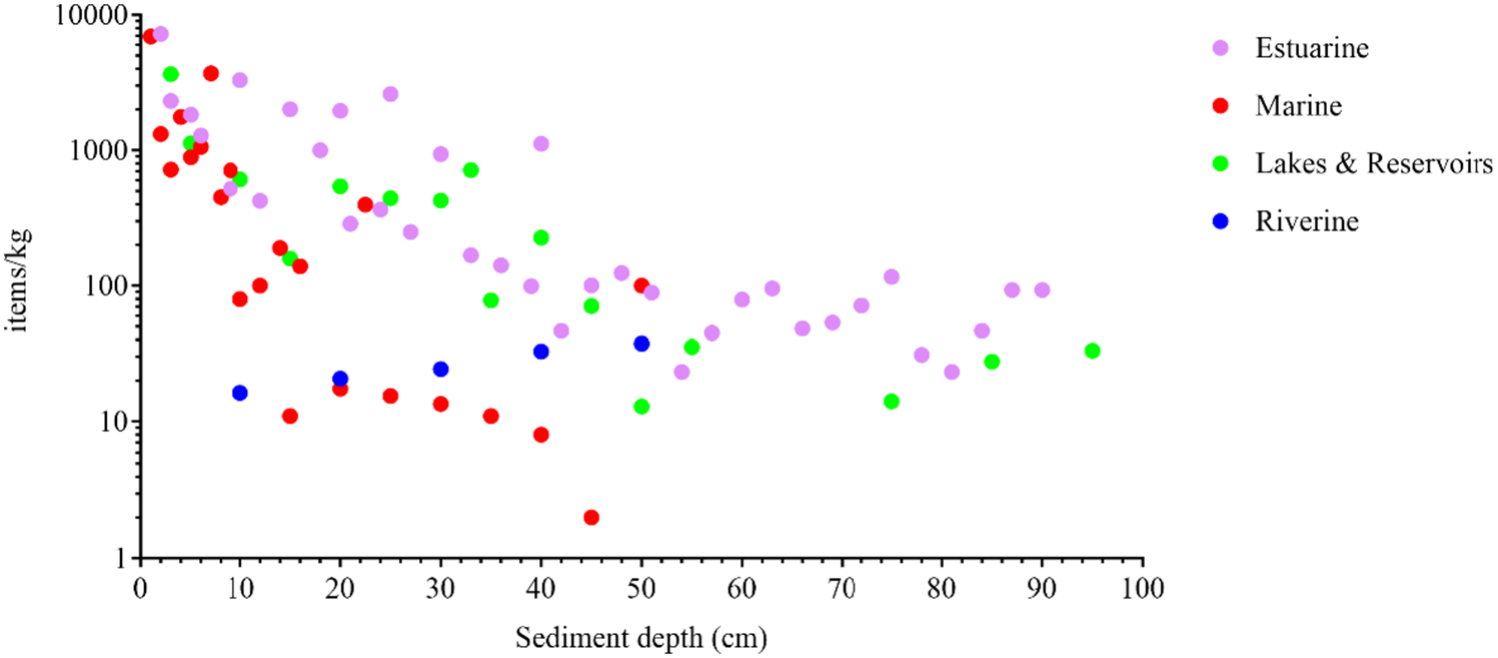
The mean microplastic concentration for each sediment layer (n = 9 studies) grouped by marine (n = 4), estuarine (n = 2), lakes and reservoirs (n = 3) and a river (n = 1). (source: MP-SED 2023)

Nine studies collected sediment cores to depths of 95 cm to investigate the temporal variation of MPs. Most cores taken from marine (Matsuguma et al. 2017; Chen et al. 2020; Zheng et al. 2020; Simon-Sánchez et al. 2022), estuarine (Matsuguma et al. 2017; Culligan et al. 2022), and lake (Turner et al. 2019; Welsh et al. 2022b; Li et al. 2023) sites demonstrated a decrease in MP concentration from surface to bottom, punctuated by sporadic peaks (Fig. 1). Irrespective of the type of water body, the uppermost 15 cm displayed the highest concentrations of MPs, showing recent accumulation or continuous inputs at these depths. In contrast, a study examining a river sediment core (0–50 cm depth; Niu et al. 2021) did not follow this trend, showing smaller and more numerous MPs in deeper layers which the authors contributed to bacterial degradation, indicating potentially different fate and deposition dynamics in fluvial systems. Niu et al. (2021) noted that the study river undergoes dredging approximately every five years, with the last event in 2015. Although the authors contribute smaller MPs in deeper layers to degradation, the dredging history highlights a potential interplay between the removal of MPs during dredging and their reintroduction from upstream sources and redistribution into the active shoaling processes. Thus, while the studies indicate a general decrease in MP concentration with depth, the process of recurrent shoaling, combined with the time scales at which it occurs, should be considered because it could result in the presence of MPs at various depths.

**Fig. 2:**
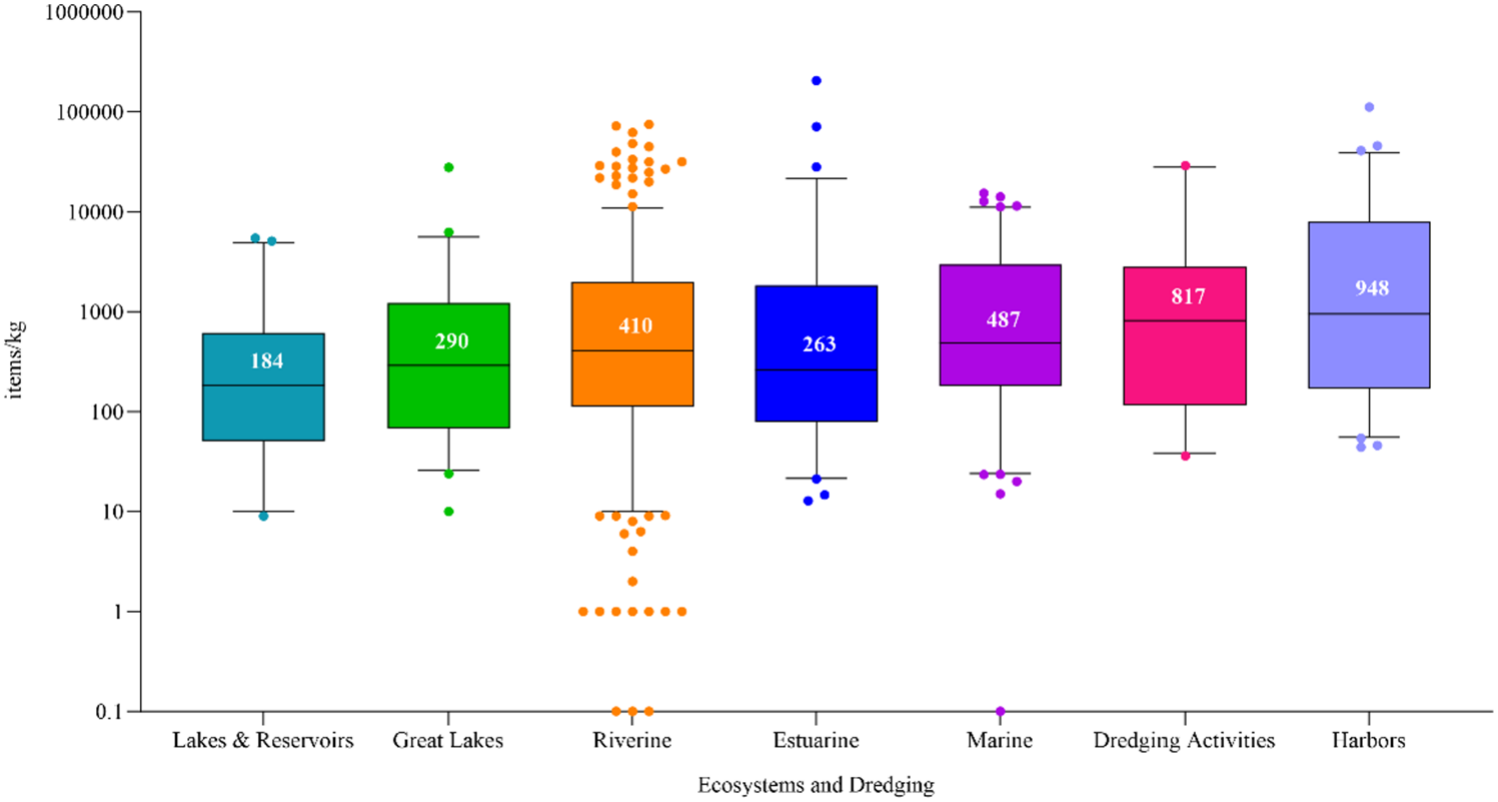
Distribution of microplastic concentrations (items/kg) reported in sediment samples. The box range = 25th to the 75th percentiles; median = horizontal line; whiskers denote the range from the 5th to the 95th percentiles. Dots indicate concentrations greater than 95th percentiles. (source: MP-SED 2023)

Four studies were identified that collected 15 sediment samples across 15 dredged sediment disposal sites and one study that collected 9 samples from sediments collected for routine evaluation prior to being dredged. The median MP concentration was 817 items/kg, ranging from 36 to 29,031 items/kg (Fig. 2). At upland dredged sediment disposal sites, Constant et al. (2021) collected sediment samples from disposal sites located along the bank of the Aa River in France, reporting MP concentrations that ranged from 1 to 2,800 items/kg which was 1 to 4 orders of magnitude lower than those in many European riverbeds. Whereas Ji et al. (2021) reported a mean of 18,911 (9,400) items/kg in dredged sediments stored in stockpiles on farmlands in China and showed that some MPs were remobilized from the piles back into surrounding soil and waters downstream. Wilkens et al. (2020) extracted MPs from sediments obtained for dredged sediment evaluations and reported a mean concentration of 1,636 items/kg for harbor sites located in the Gulf of Mexico, Atlantic Ocean, and Great Lakes. Notably, the highest MP reported in that study came from the Cleveland Harbor (5,019 items/kg) which is an area that experienced severe industrial pollution in the past (e.g., fires on the Cuyahoga River).

Eight studies collected 42 surficial sediment samples across14 lakes where MPs were omnipresent. The median MP concentration was 184 items/kg, ranging from 9 to 5450 items/kg. The rural headwater lakes in Muskoka–Haliburton, Ontario, recorded the highest concentrations, where fibers were the most prevalent type, suggesting atmospheric deposition as a significant source, as supported by Welsh et al. (2022a, 2022b). Conversely, Vaughan et al. (2017) collected sediment from an urban lake, which might be expected to have higher concentrations due to greater local human activity but exhibited some of the lowest MP concentrations. In another context, Wilkens et al. (2020) reported 1114 items/kg in an oxbow lake along the Mississippi River which is annually inundated by the river estimated to contribute more than five quadrillion MPs into the Gulf of Mexico annually (Martin 2018). The data reveal a substantial range in MP concentrations across rural and urban lakes, suggesting that their complex presence and distribution are influenced by varied factors like atmospheric deposition and river connectivity, pointing to multiple sources and transport mechanisms beyond human proximity.

Four studies that collected surficial sediment samples from tributaries and nearshore areas in the Great Lakes, covering Lake Michigan (n = 9), Lake Erie (n = 15), Lake Huron (n = 7), and Lake Ontario (n = 26), all found microplastics (MPs). Across these studies, the median MP concentration was 290 items/kg, with a range from 10 to 27,830 items/kg. Breaking this down further, tributaries had a median MP concentration of 370 items/kg, ranging from 10 to 27,830 items/kg, while nearshore areas had a median of 285 items/kg, with a range from 24 to 5,530 items/kg. Urban tributaries, in particular, showed elevated MP concentrations. For instance, Ballent et al. (2016) found a mean MP concentration of 4,500 (± 10,308) items/kg in Lake Ontario tributaries passing through urban areas, which was substantially higher than the 567 (± 459) items/kg found in nearshore samples. Likewise, Lenaker et al. (2019) reported a mean MP concentration of 2,034 (± 2,492) items/kg in Lake Michigan tributaries through urban areas, which exceeded the mean of 197 (± 116) items/kg for nearshore samples. These findings underscore the significant role of urban tributaries as contributors to MPs in the Great Lakes system, aligning with observations that rivers and streams are key pathways for MP contamination (Talbot and Chang 2022).

Thirty papers collecting 429 surficial sediment samples across 75 rivers, all contained MPs except for three samples. The median MP concentration was 410, ranging from 0 to 74,800 items/kg. Notably, Wang et al. (2018) found the highest concentration of 32,947 (range 18,690 to 74,800) items/kg in the Wen-Rui Tang River in China, an area with high levels of industrial activity. Hurley et al. (2018) also reported elevated mean MP concentrations of 31,950 (range 1,700 to 62,200) items/kg in River Glossop Brook and 21,300 (range 500 to 72,400) items/kg in River Tame in the UK. Additionally, in the same study Hurley et al. (2018) noted a decline in MP concentrations in 28 out of 40 river sites following a major flood event (mean pre-flood 7,036 items/kg; post-flood 889 items/kg), showing that MP contamination can substantially change following flooding. Eppehimer et al. (2021) found similar results for MP concentrations after a flood in the Santa Cruz River near Tucson, Arizona.

Twelve studies collecting surficial sediment samples from 12 nearshore marine areas (n = 106 samples) and 15 studies collecting surficial sediments from 17 estuarine areas (n = 72 samples) environments, all contained MPs except for one marine site. The overall median MP concentrations within marine environments was 487 items/kg, ranging from 0 to 15,326 items/kg and within estuarine environments was 263 items/kg, ranging from 13 to 205,859. In marine nearshore areas, concentrations were reported with a mean of 9,510 (± 3,209) items/kg in the Arabian Sea along the Indian coast by Gurjar et al. (2023), and 2,866 and 3,359 items/kg in the Celtic and North Seas, respectively, by Bakir et al. (2023). In estuarine systems, Haave et al. (2019) reported 79,301 items/kg in a Norwegian fjord near a wastewater facility. A standout report from Cashman et al. (2022) documented an extreme mean concentration exceeding 5.26 million items/kg, dominated by cellulose acetate fibers, in Narragansett Bay, USA, but was excluded from the data set since the MP concentrations were at least two orders of magnitude higher than other locations.

Seven studies collecting 68 surficial sediment samples across 18 harbor locations, all contained MPs. The median MP concentration was 948 items/kg, ranging from 44 to 111,933 items/kg. The results were skewed by the highest concentrations (n = 22) all reported by Preston-Whyte et al. (2021) for sediments collected from the Port of Durban with a mean MP concentration of 21,067 (range 2,400 to 111,933) items/kg. The authors suggested that sewage overflow, stormwater drains, port operations, and rivers were primary MP contributors. Excluding the Port of Durban, the median MP concentration for the remaining samples (n = 46) decreased to 237 items/kg. The data reveal a substantial range in MP concentrations across harbor areas, suggesting again that their complex presence and distribution are influenced by varied factors like atmospheric deposition, river inputs, and harbor activities.

Regulatory agencies, public health officials, scientists, and the public are increasingly concerned with MPs in the environment due to their potential implications on human health, regulatory frameworks, and environmental ecosystems. Researchers have begun to explore complex toxicological datasets to formulate risk-based MP thresholds for ecological receptors in sediment. These risk-based thresholds, reflective of an evolving confidence (Mehinto et al. 2022), are specific to two anticipated mechanisms of action observed during laboratory toxicity evaluations: 1) food dilution, where MPs are mistakenly ingested in place of more nutritive food items (Straub et al. 2017; de Ruijter et al. 2020; Rauchschwalbe et al. 2021); and 2) the translocation of MPs into tissue, resulting in inflammation and oxidative stress (Limonta et al. 2019; Xia et al. 2020). In sediment, risk-based thresholds for food dilution range from 6.6 × 10^7^ items/kg to 1.9 × 10^11^ items/kg, and for the translocation of MPs in tissues, 3.2 × 10^8^ to 4.0 × 10^11^ items/kg (Redondo-Hasselerharm et al. 2023). As such, current reported MP concentrations in sediments typically fall below these threshold values. However, ongoing research is crucial for refining these thresholds, including incorporating the complexity of MPs and considering them alongside natural particles in the environment (Koelmans et al. 2022), to fully understand the implications.

This review presents initial findings on the presence of MPs in sediments that may be relevant to dredging programs, with the highest median concentration found in harbor sediments. Notably, MPs are less prevalent in deeper, undisturbed sediment layers targeted by capital dredging compared to the recurrent shoaling sediment of maintenance dredging. The high variability at different depths and environments highlights the ubiquitous nature of MPs–both in areas where dredging occurs and in places where it does not. This variation highlights the critical need for proactive measures at the source to prevent plastic contamination from entering sediments. These preventative strategies could include improved wastewater treatment, stormwater controls, and supply chain modifications. Monitoring MPs in sediments, including prospective dredged sediments, is a potentially important tool for assessing the effectiveness of preventative strategies. Since the 1970s, in the United States, dredged sediment proposed for open water disposal undergoes rigorous evaluation and testing to ensure it does not adversely affect human health or the environment (USEPA and USACE 1991). This often involves bioassays capable of detecting the cumulative toxicity of chemical mixtures, both known and *unknown*, compared to a reference sediment. Although the recognition of MPs in dredged sediments is relatively recent, their potential contribution to cumulative toxicity continues to be assessed through these thorough evaluations. Despite the number of manuscripts pertaining to MPs in sediment, key data gaps remain. Research is needed to better understand ecotoxicological effects of environmentally relevant MP exposures. A critical component toward improving this understanding involves comprehensively documenting MP characteristics such as size, morphology, polymer type, and toxicity. Collating this information in resources like the MP-SED and ToMEx (Thornton Hampton et al. 2022) databases is important to help inform future risk assessments for plastics in sediments.
